# Supplementary figures and images for: Genome-Wide Identification of PYL/RCAR ABA Receptors and Functional Analysis of LbPYL10 in Heat Tolerance in Goji (Lycium barbarum)
Source: Plants (Basel). 2024 Mar 20;13(6):887. doi: 10.3390/plants13060887 (PMC10975129; doi:10.3390/plants13060887)

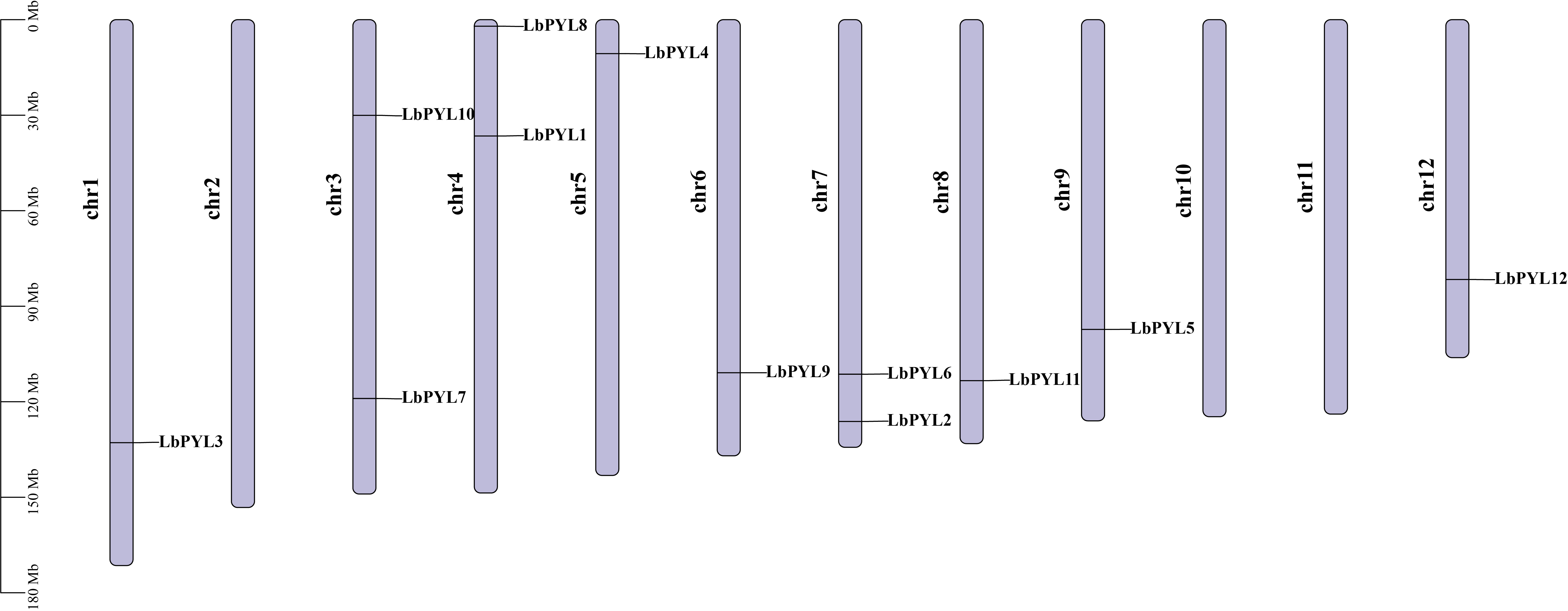

Supplement: Supplementary file 1 [file plants-13-00887-s001.zip › Figure S1. Chromosomal distribution of LbPYLs genes..jpg]

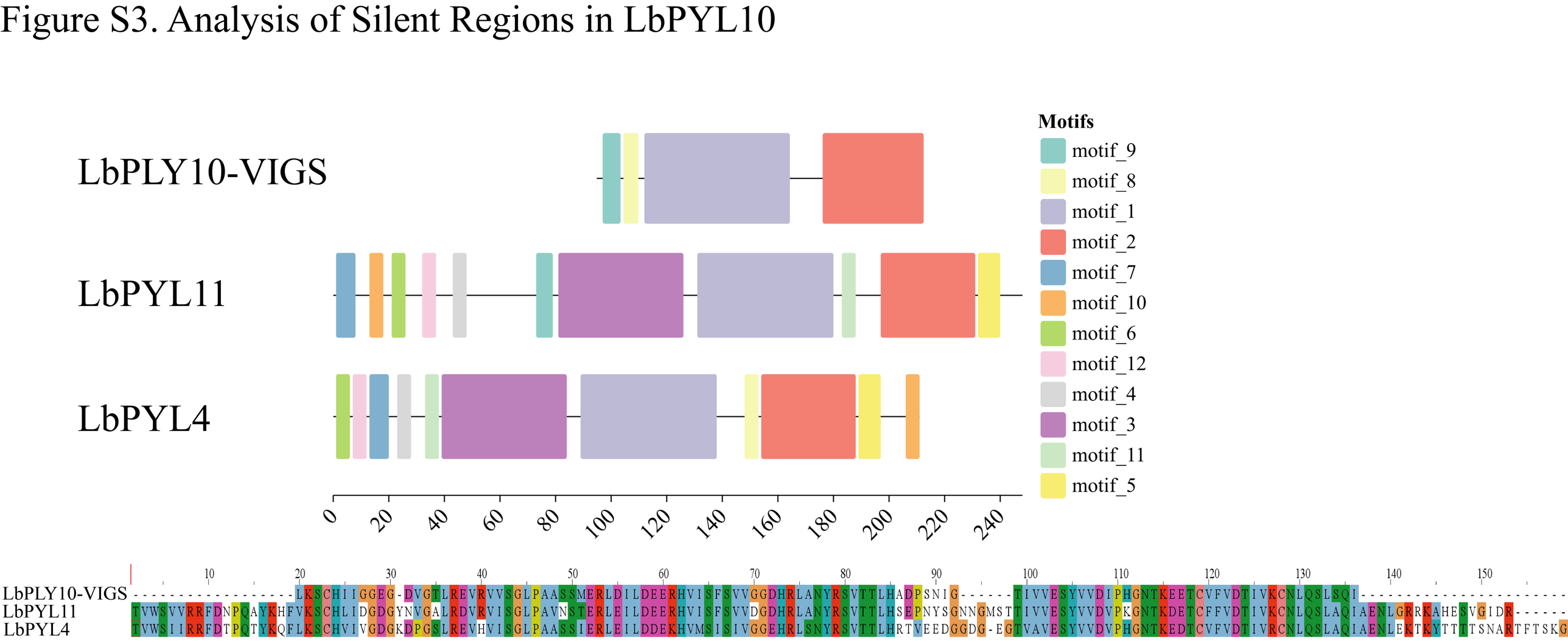

Supplement: Supplementary file 1 [file plants-13-00887-s001.zip › Figure S2.Analysis of Silent Regions in LbPYL10.jpg]

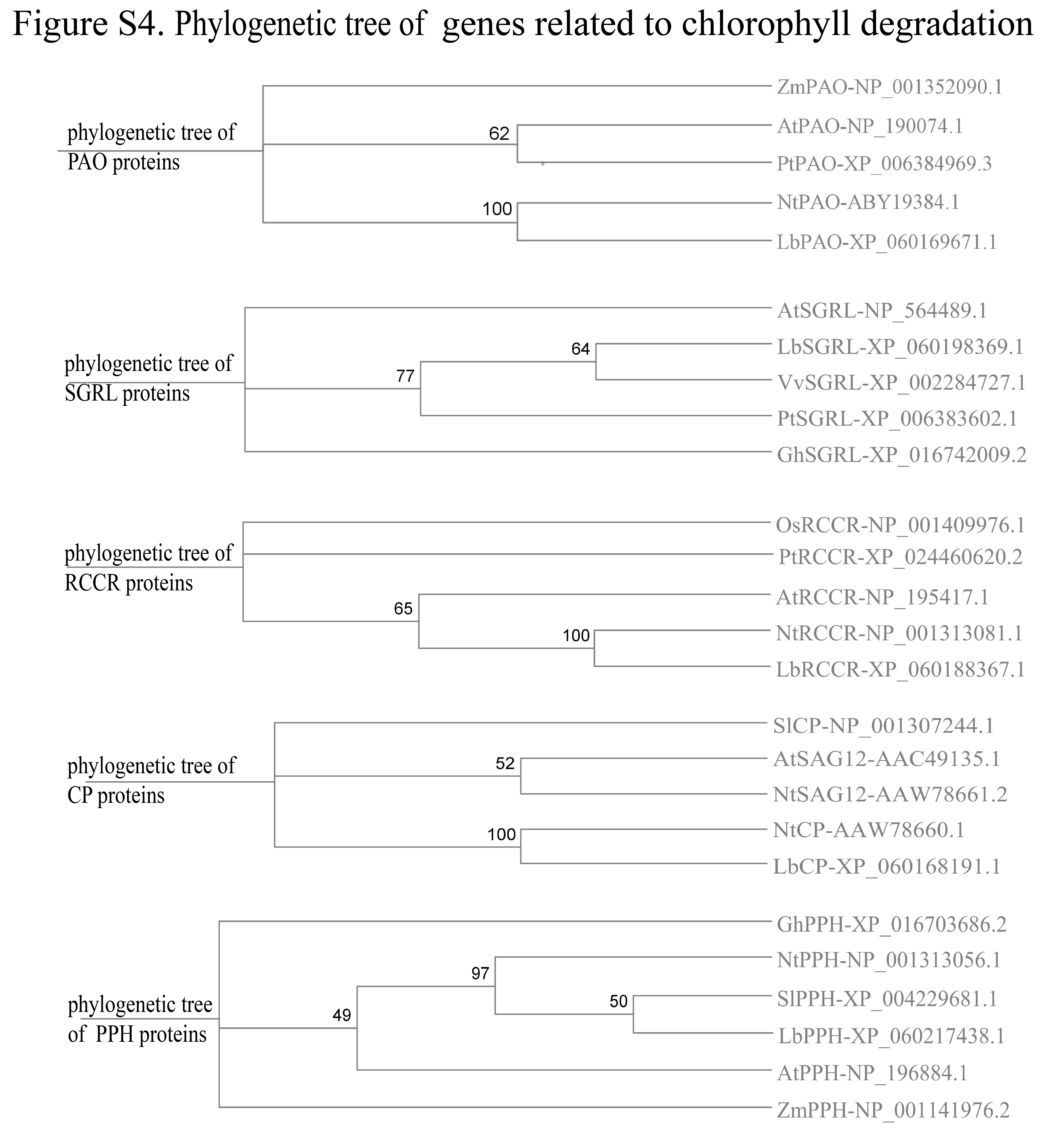

Supplement: Supplementary file 1 [file plants-13-00887-s001.zip › Figure S3. Phylogenetic tree of genes related to chlorophyll degradation.jpg]

Figure S2. Conserved domain in PAO and SGRL proteins

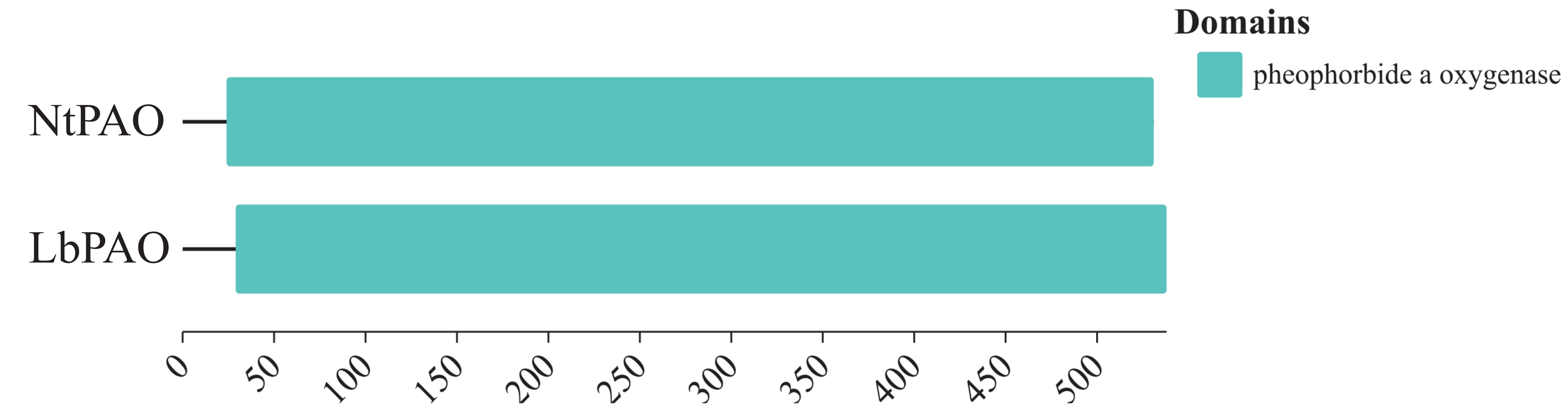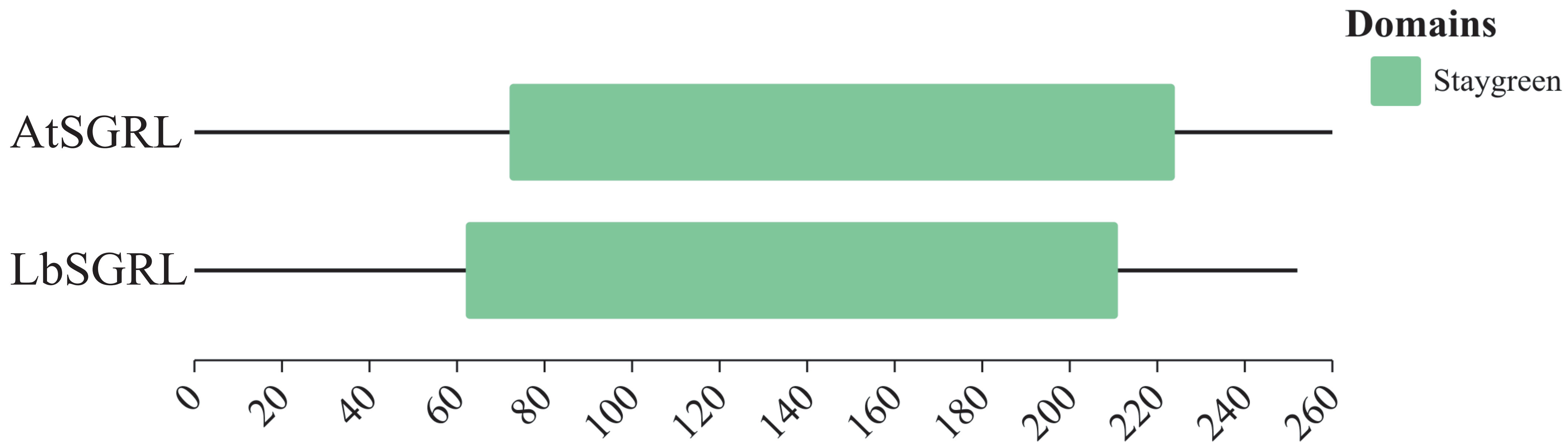

Supplement: Supplementary file 1 [file plants-13-00887-s001.zip › Figure S4.Conserved domain.pdf]

Figure S1. Conserved motifs in PAO and SGRL proteins

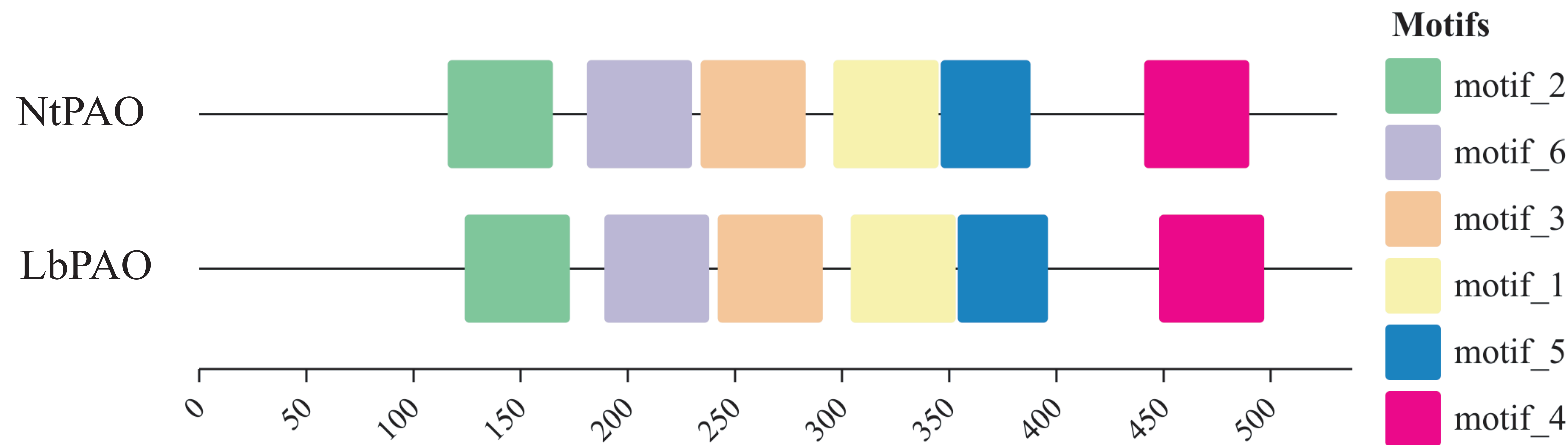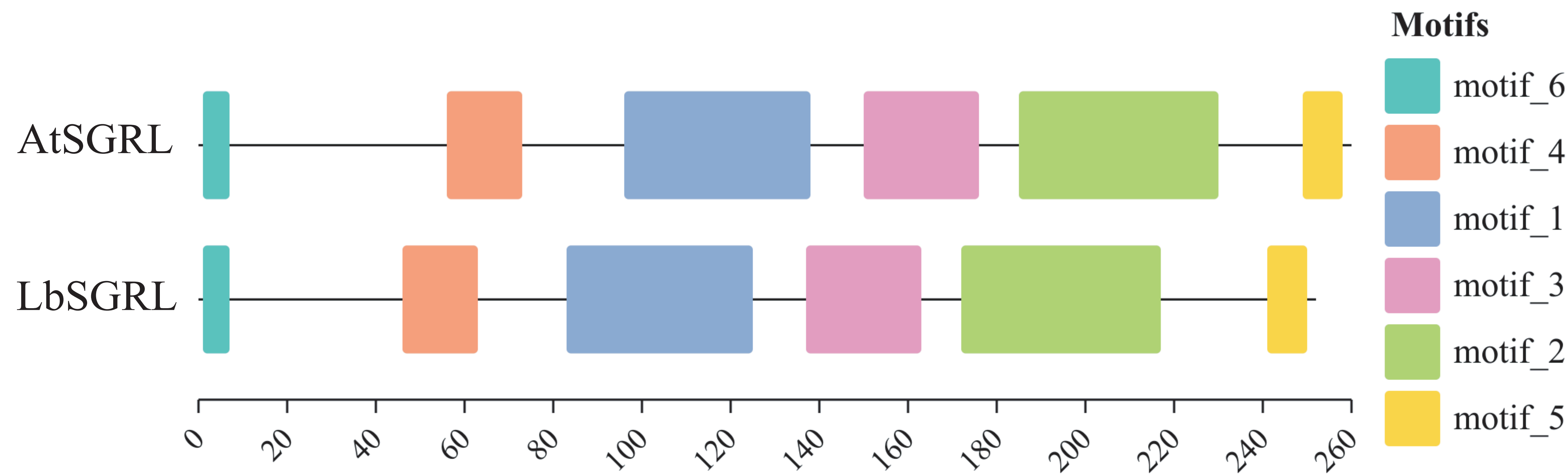

Supplement: Supplementary file 1 [file plants-13-00887-s001.zip › Figure S5.Conserved motifs.pdf]
